# Pharmacokinetic Analysis of Zonarol, a Marine Algal Hydroquinone, in Mice Using HPLC with Fluorescence Detection

**DOI:** 10.3390/antibiotics12061013

**Published:** 2023-06-05

**Authors:** Jiyao Fei, Sohsuke Yamada, Takumi Satoh, Tomoyuki Koyama

**Affiliations:** 1 Graduate School of Marine Science and Technology, Tokyo University of Marine Science and Technology, Tokyo 108-8477, Japan; 2 Department of Pathology and Laboratory Medicine, Kanazawa Medical University, Ishikawa 920-0293, Japan; sohsuke@kanazawa-med.ac.jp; 3 Department of Anti-Aging Food Research, School of Bioscience and Biotechnology, Tokyo University of Technology, Tokyo 192-0982, Japan; satotkm@stf.teu.ac.jp

**Keywords:** zonarol, pharmacokinetics, brown algae *Dictyopteris undulata*, hydroquinone, blood–brain barrier, mice

## Abstract

Zonarol, which was discovered in the brown algae *Dictyopteris undulata,* has antibiotic, antioxidative, anti-inflammatory, and neuroprotective hydroquinone properties. Additionally, a daily treatment of zonarol taken orally has been proven to prevent ulcerative colitis and nonalcoholic fatty liver disease in experimentally induced mice models. In this study, to elucidate the physiological behavior of zonarol in vivo, the establishment of quantitative methods for the determination of zonarol in biological samples and basic pharmacokinetics parameters after oral or intravenous administration with purified zonarol to mice were investigated. The zonarol (20–600 ng/mL) in this study was dose-dependently detected using an HPLC-FI system as a single peak on the ODS column with 80% aqueous methanol at 332 nm with an excitation of 293 nm. The pharmacokinetic parameters were derived from a non-compartment analysis of the plasma concentration of zonarol following oral or intravenous treatment in mice. The absolute bioavailability of zonarol was calculated as 25.0%. Interestingly, the maximal distribution of zonarol in the brain (2.525 ± 1.334 µg/g tissue) at 30 min was observed to be higher and slower than that in the liver and kidney at 15 min after bolus intravenous administrations to the mice (10 mg/kg BW). Based on these results, zonarol might be a candidate for a potential drug, an effective tool for drug delivery, or enhancing the treatment of cerebral disease.

## 1. Introduction

Marine species comprise approximately half of total global biodiversity [1]. Seaweeds are extensively consumed in East and Southeast Asian countries, including Korea, Japan, and parts of China [2]. Seaweeds are rich sources of bioactive compounds with various biological activities [3]. Recently, researchers have revealed that seaweed compounds also exhibit various biological activities [4,5,6,7].

Brown algae are a group of multicellular heterokonts that are ubiquitous in today’s oceans [8] and whose chemistry is dominated by terpenoids and phenolics [9]. The brown algae *Dictyopteris undulata* belong to the family Dictyotaceae, and they are commonly found in East Asian countries, such as Japan, Korea, and China. In addition, they have been reported to have many biological activities, such as the induction of apoptosis in cancer cells [10,11], lysis activity [12], antioxidative activity [13], etc.

Zonarol, a sesquiterpene hydroquinone that is separated from the methanol extract of the brown algae *Dictyopteris undulata*, was first reported in 1973 as a fungitoxic compound [14]. However, more pharmacological actions of zonarol have been subsequently reported, such as phospholipase inhibition [15] and algicidal effects [12]. Recently, the anti-inflammatory activity and the anti-oxidative activity of zonarol have been widely researched. Yamada et al. revealed that zonarol produced anti-inflammatory activities in dextran-sulfate-sodium (DSS)-induced ulcerative colitis in mice as it inhibited the activation of macrophages and also inhibited the expression of certain inflammatory cytokines [16]. Shimizu et al. reported that zonarol provides neuroprotective activity by activating the Nrf2/ARE pathway and by increasing the expressions of anti-oxidative enzymes, i.e., the “phase-2 enzymes”, including NADPH quinone oxidoreductase 1 (NQO1), heme oxygenase-1 (HO-1), and peroxiredoxin 4 (PRDX4) [17]. Han et al. found that zonarol protects the liver from nonalcoholic liver disease (NAFLD) in mice by reducing inflammatory cell infiltration, hepatic lipid deposition, and oxidative stress [18]. Based on these pieces of evidence, zonarol may be a promising candidate for drug development with its potential pharmacological activity against many lifestyle-related diseases. However, the absorption and pharmacokinetics of zonarol in vivo are still unclear. Without this information, it is difficult to quantify the pharmacological effects of zonarol, and it is also difficult to identify the specific sites in the body where it can exert its influence.

In this study, for the first time, the plasma concentration of zonarol in mice was measured after oral administration (p.o.) or intravenous administration (i.v.) in order to detect the time-dependent changes. Additionally, the tissue distribution of zonarol was also measured after intravenous administration.

## 2. Results and Discussion

### 2.1. Fluorescence Detection of Zonarol Using HPLC

**Figure 1:**
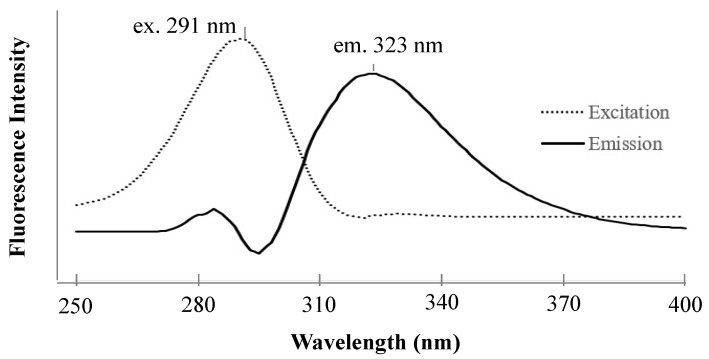
The excitation and emission spectra for 1 μM of zonarol in methanol.

The fluorescence spectra of zonarol (1 µM) are shown in Figure 1. The maximum excitation and emission wavelengths of zonarol were observed at 291 nm and 323 nm, respectively. These spectra were similar to that of the tetra butyl hydroquinone (TBHQ) in MeCN [19]. The fluorescence conditions for detecting zonarol were defined as the same as those for the measurement of TBHQ (ex. 293 nm and em. 332 nm) in the literature above. The common conditions to detect hydroquinones, including the metabolites of zonarol in vivo, in future studies will be the same as those used in the same HPLC analysis in this study.

**Figure 2:**
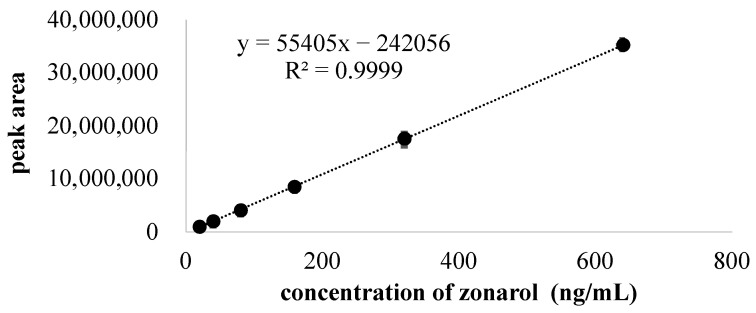
The standard curve and regression equation of zonarol. Zonarol was dissolved with methanol and the concentrations were 20, 40, 80, 160, 320, and 640 ng/mL. Data are expressed as the mean ± S.D. (*n* = 3).

The standard curve of zonarol that was created via fluorescence detection is shown in Figure 2. The linear regression equation of the peak area (y) vs. zonarol concentration (x) was y = −202,456 + 55,405 x, with a correlation coefficient of 0.9999. The limit of quantification (LOQ) of the zonarol was verified as 11.1 ng/mL at a signal-to-noise ratio of 10. The limit of detection (LOD) of the zonarol was verified as 6.41 ng/mL at a signal-to-noise ratio of 3. The high correlation coefficient and the low LOQ and LOD suggest that the HPLC condition was accurate and sensitive.

### 2.2. Intra-Day and Inter-Day Repeatabilities of Zonarol Detection

The results of the intra-day and inter-day detections are shown in Table 1. The precision (RSD) was lower than 5.3%. The accuracies of intra-day detection were in a range of 95.9–107.8%. The accuracies of inter-day detection were in a range of 94.7–108.5%. These results indicated that the HPLC condition demonstrated acceptable accuracy and precision.

### 2.3. Stability of Zonarol with Plasma during HPLC Analysis

The short-term stability of zonarol in the mice plasma under different conditions are shown in Table 2. The mean relative standard deviation (RSD) was lower than 6.7%, which suggests that the HPLC condition was accurate for the detection of zonarol in plasma. The accuracy of each group was at a range of 90.8–112.9%, which indicates that the zonarol was stable in plasma. The results show that the zonarol content did not change when stored at RT or 37 °C for 4 h. The subjection of the zonarol to three freeze–thaw cycles appeared to have no effect on the quantification of the analyte. The zonarol stored at 4 °C remained stable for at least 12 h. These results suggest that zonarol will not undergo spontaneous change during HPLC analysis. On the other hand, the stability of zonarol in longer storage conditions or in the body must be evaluated in more various conditions in the near future.

**Figure 3:**
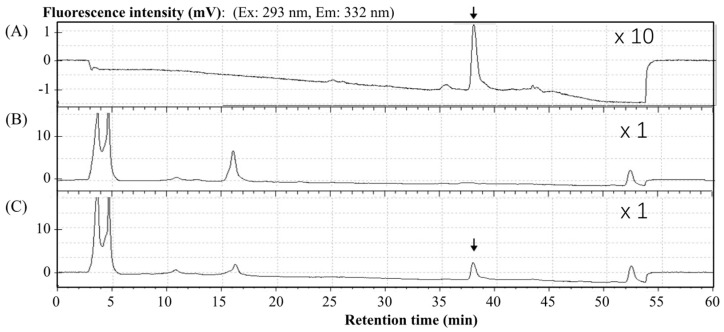
Representative chromatograms of (**A**) a zonarol sample (80 ng/mL), (**B**) blank plasma, and (**C**) plasma spiked with zonarol (80 ng/mL). The position of the peak of zonarol is marked with an arrow in (**A**,**C**).

Representative chromatograms are shown in Figure 3. Under the HPLC conditions detailed above, the zonarol was eluted with a retention time of 37.6 min. The innate components in the mice plasma (B) did not produce any interfering peaks. In the chromatogram of the plasma spiked with zonarol (C), the peak derived from zonarol can be clearly detected. Meanwhile, the peak of sufficient zonarol can be detected and quantified in plasma.

On the other hand, as xenobiotics are oxidized and/or conjugated by enzymes during circulation in vivo, certain metabolites of zonarol circulate within the blood stream. The important candidates for this were the oxidized form, i.e., the quinone form known as zonarone, which is assumed to be the key metabolite that contributes to anti-oxidative activity [17], and the conjugated form with sulfate and glucuronide by phase 2 enzymes [20]; however, the fluorescence detection method specialized to hydroquinone has insufficient sensitivity to detect these metabolites. A detailed understanding of these metabolites requires further continuous studies that relate to metabolite and excretion routes. In the current research, to elucidate on the bioactivities in vivo, the intact form of zonarol was focused upon as an important active form.

### 2.4. Time-Course Changes in Plasma Concentrations of Zonarol after Oral or Intravenous Administration in Mice

**Figure 4:**
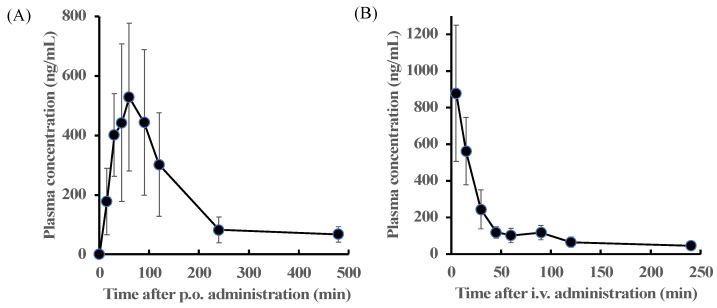
Time-dependent changes in the plasma concentration of zonarol (**A**) after oral (100 mg/kg, p.o.) (*n* = 5, mean ± SD) or (**B**) intravenous administration (10 mg/kg, i.v.) (*n* = 6, mean ± SD).

This section details the methods that were used for determining the plasma concentration of zonarol following oral (100 mg/kg) or intravenous administration (10 mg/kg). The time-dependent courses of the mean plasma concentration of zonarol are shown in Figure 4. The pharmacokinetic parameters were calculated and fitted in a non-compartment model and are summarized in Table 3. After oral administration, the plasma concentration of the zonarol reached its highest at 75 ± 13.41 min. Meanwhile, the maximal concentration was 722.51 ± 75.93 ng/mL, and the zonarol still remained over the LOQ within 480 min. The plasma concentration of zonarol was maintained at a relatively high level within 120 min after oral administration. On the other hand, the plasma concentration of zonarol at 5 min after intravenous administration was 878.47± 371.16ng/mL. Then, it declined rapidly; however, it still remained over the LOQ within 240 min. The values of the area under the curve from 0 to 480 min (AUC_0-480_) and from 0 to 240 min (AUC_0-240_) were 91,333.84 ± 31,976.29 and 36,470.64 ± 5734.82 min × ng/mL for the p.o. and i.v. experiments, respectively. The bioavailability of the zonarol in mice was 25.0%.

After oral administration, the zonarol quickly reached its maximum concentration, which was considered to be due to its high hydrophobicity and low molecular weight [20]. However, the highest concentration of zonarol after oral administration was still relatively low compared to that found in the other terpenoids and phenolic compounds reported in food materials, such as carnosic acid, a different pro-electrophilic compound [21,22]; carotenoids; and flavonoids [23,24,25]. For example, the absolution rate of quercetin was affected by enterohepatic recirculation [24,25,26]; in addition, the absolute bioavailability was 16%, and its variability depended on the condition of its gastric contents during ingestion [27]. Additionally, fucoxanthin, a major carotenoid in brown algae, has also shown antioxidative and neuroprotective properties in vivo; however, its bioavailability was reported as 0.06% [28].

There are several reports that have described the bioactivities that occurred in mice following the oral administration of zonarol. In our previous reports, an oral bolus administration of zonarol (62.5 mg/kg) in mice at −0.5 h significantly suppressed carrageenan-induced paw edema at 2 and 4 h after carrageenan treatment at 0 h [16]. Based on the current results following the oral administration of zonarol (100 mg/kg), approximately 300 ng/mL of zonarol in plasma was required to induce suppressive effects on edema formations in mice. On the other hand, the oral administration of zonarol (10 mg/kg/day) in the experimental mice model for 2 weeks suppressed DSS-induced ulcerative colitis [16] and prevented diet-induced NAFLD [18]. The current results obtained with a bolus administration of zonarol cannot estimate the dynamics of the active compound. Further pharmacokinetic data in experiments with chronical and/or multiple administrations of zonarol are needed to better understand its dose–response relationship in vivo.

### 2.5. Tissue Distribution of Intravenously Administered Zonarol

**Figure 5:**
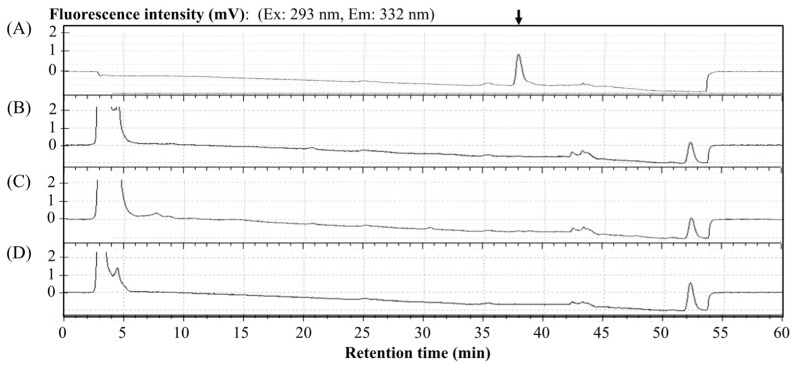
The representative chromatograms in aliquot from blank tissue homogenates without zonarol that were used to confirm the baseline around the peak position of zonarol: (**A**) zonarol solution (80 ng/mL) as standard, (**B**) blank liver homogenate, (**C**) blank kidney homogenate, and (**D**) blank brain homogenate. The peak position of zonarol is marked with a vertical arrow in (**A**).

In advance of measuring the zonarol contents in the tissues, a baseline chromatogram with the control aliquot extracted from the tissue homogenates without zonarol was confirmed by the HPLC methods detailed above. As is shown in Figure 5, the peak position of zonarol (80 mg/mL) is shown with an arrow in (A) and was not interfered with by any unspecific noises or components that may derive from the homogenates of the liver (B), kidney (C), and brain (D).

**Figure 6:**
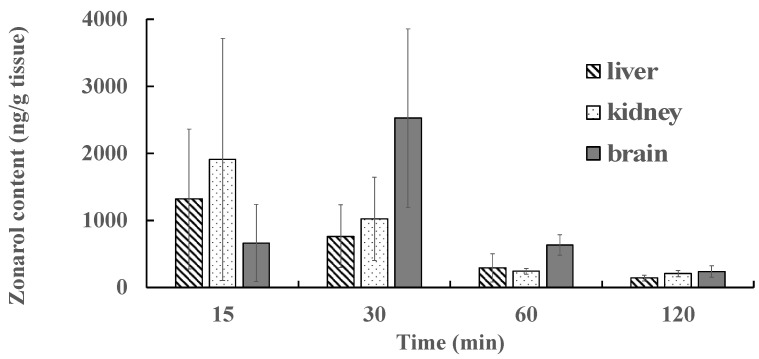
Distribution of zonarol in the various tissues (the liver, kidney, and brain) at different times after intravenous administration (10 mg/kg) (*n* = 7). Data are expressed as the mean ± SD of the zonarol content (ng) per tissue weight (g).

The tissue/body weights (g/g) and the visual observation of each tissue did not reflect any marked differences between the groups with zonarol, either with or without bolus treatment (data are not shown). The results of the tissue distribution after the intravenous administration of zonarol are summarized in Figure 6. The results obtained after the intravenous administration of zonarol at 15, 30, 60, and 120 min in various tissues (liver, kidney, and brain) are shown in Figure 6. The data are expressed as the zonarol content (ng) per tissue weight (g) for each of these experiments. In the cases of the liver and kidney at 15 min after administration, the highest content of zonarol was shown as 1320.2 ± 1042.4 ng/g and 1911.3 ± 1803.6 ng/g, respectively. On the other hand, in the case of the brain, the peak of the content, which appeared at 30 min after administration, was 2525.5 ± 1333.7 ng/g tissue at 30 min. The zonarol levels in all three of the tissues were suppressed at 120 min after administration.

Interestingly, the accumulating content of zonarol in the brain was relatively higher and slower than those found in the liver and kidney. These results suggest that the hydroquinone passes through the blood–brain barrier (BBB) via passive diffusion, which occurs due to the hydrophobicity and/or active transport systems that operate via specific mechanisms. Based on certain reports on the health-promoting compounds in food, a 0.1%-quercetin-containing diet for 11 weeks did not induce the accumulation of quercetin in the brain [29]. In a chronic study with carnosic acid in rats, the accumulating contents in both the liver and the brain showed similar trends to each other [30]. Fucoxanthin, a popular carotenoid found in brown algae, has also been known to pass through the BBB due to its hydrophobic properties [28,31]. A common property of fucoxanthin and zonarol is their distribution rate in the brain. However, the absolution rate of carotenoids in blood after oral administration is quite a bit lower than that of zonarol. These unique properties of zonarol will serve as an advantage for applying treatment or for delivery targeting to the brain. As the spiking test of zonarol with each tissue homogenate has not been performed, the effects of tissue-dependent components could not be rejected. Further chronic and metabolite studies with zonarol will help to understand its potential applications. Moreover, zonarol could be a potential drug or drug delivery agent for treating cerebral disease in the future.

## 3. Materials and Methods

### 3.1. Algal Materials and Chemicals

The brown algae *Dictyopteris undulata* were collected on a rocky seashore in the Kanto Region, Japan, and kept in a refrigerator at −30 °C until their use in experiments. Zonarol was extracted and purified from the algal materials according to the procedure detailed in our previous report [16], which is described briefly in Appendix A. The structure and purity of zonarol were confirmed by ^1^H NMR analysis as shown in Appendix B.

For the experiments in the current report, guaranteed-grade and/or HPLC-grade organic solvents, e.g., acetonitrile (MeCN), methanol (MeOH), ethyl acetate (EtOAc), hexane (Hex), dimethyl sulfoxide (DMSO), formic acid, and distilled water, were purchased from Kanto Chemical Co., Inc (Tokyo, Japan). Silica gel (0.063–0.200 mm, Merck KGaA, Darmstadt, Germany) and ODS gel (Nacalai Tesque Inc., Kyoto, Japan) were used for column chromatography. Sterile saline solution (0.9%) was obtained from Otsuka Pharmaceutical Co., Ltd. (Tokyo, Japan).

### 3.2. Preparation of Zonarol Solution for In Vitro and In Vivo Experiments

The primary stock solutions of purified zonarol were prepared at concentrations of 1 mg/mL MeOH. This primary stock solution was divided into small tubes and stored at −20 °C until its use in all experiments in the current research.

To confirm the fluorescence activities, a MeOH solution of zonarol at a concentration of 1 μM was prepared separately. The fluorescence spectra, with their exciting and emission wavelengths, were measured with an RF-1500 (Shimadzu, Kyoto, Japan).

The zonarol solution for oral administration was prepared by dissolving zonarol in corn oil less than 1 h before the experiment. The zonarol solution for intravenous administration was prepared by dissolving zonarol in a 5% DMSO-normal saline solution.

### 3.3. HPLC Condition

HPLC analysis was carried out on a Shimadzu-20A series instrument (Shimadzu, Japan) connected to a fluorescence detector (RF-10A xl, Shimadzu, Japan). The signals from the fluorescence system were transmitted to LC Solution software for the purposes of analyzing and calculating the peak areas. Chromatographic separation was carried out on an RPAQUEOUS-AR-5 (4.6 × 250 mm) column. The HPLC was operated with a gradient mobile phase system consisting of water containing 0.1% formic acid (phase A) and methanol (phase B) at a flow rate of 1 mL/min. The pump was programmed as follows: phase B was maintained at 40% for the first 5 min, increased from 40% to 80% within the next 25 min, maintained at 80% for the next 10 min, increased to 100% for 10 min, and then decreased back to 40% for 10 min (total gradient time: 60 min). A 50 μL sample was injected into the system with the auto-sampler and conditioned at 4 °C, and the column temperature was maintained at 30 °C.

The standard curve was constructed in the concentration range of 20–640 ng/mL of zonarol, which was dissolved with methanol by plotting the peak-area (y) of the zonarol against the concentration of zonarol (x). Zonarol samples were analyzed in triplicate. The regression parameters of slope and correlation coefficient were also calculated.

### 3.4. Repeatability of Zonarol Detection

The accuracy and precision (presented as relative standard deviation (RSD)) of the analytical method were evaluated using zonarol samples, which were prepared as described above. The intra-day precision was determined at low, medium, and high concentrations (20, 80, and 320 ng/mL) six times a day. The same procedure was performed once a day for three consecutive days to determine the inter-day precision. The accuracy of the assay was determined by comparing the nominal concentrations with the corresponding calculated concentrations via linear regression.

### 3.5. Animals and Preparation Biological Samples

Male ddY mice (8 weeks old, 40 ± 2 g) were purchased from SLC Inc. (Shizuoka, Japan) and were acclimated to laboratory conditions for 1 week before experiment. The mice were housed in an air-conditioned room at 23 ± 2 °C with 12 h/12 h light–dark illumination cycles at a constant humidity (55 ± 10%) with free access to food (MR stock, Nosan, Japan) and tap water. Exsanguination and autopsy were performed under anesthesia with an overdose of isoflurane (Mylan EPD Ltd., Canonsburg, PA, USA). All procedures were reviewed and approved (R3-9 and R4-2) in terms of animal welfare by the Animal Ethics Committee of Tokyo University of Marine Science and Technology.

Whole blood was taken from the abdominal vein of the mice after anesthetization. The whole blood in heparinized tube was immediately centrifuged at 9000 rpm for 5 min after being collected. Then, the supernatant (plasma) was collected. Blank plasma was stored at −20 °C until used. Plasma samples were obtained by centrifuging whole blood, which was taken from the abdominal vein of mice 30 min after the oral administration of 100 mg/kg zonarol dissolved with corn oil, at 9000 rpm for 5 min.

### 3.6. Feasibility Studies to Detect Zonarol in Biological Samples

The feasibility of using this HPLC condition to detect zonarol was determined by preparing a zonarol solution dissolved in MeOH, a blank plasma sample, a plasma sample spiked with zonarol, and a pharmacokinetic plasma sample from mice after oral administration.

The stability of low, medium, and high concentrations of 20, 80, and 320 ng/mL of zonarol mixed with the plasma of mice was performed under different conditions, including storage at room temperature for 4 h, storage at 37 °C for 4 h, and storage at 4 °C for 12 h and subjection to three freeze–thaw cycles. Next, 20 μL aliquots of low, medium, and high concentration zonarol solutions (16, 4, and 1 μg/mL) were added to polypropylene tubes with 80 μL of blank plasma. Then, 900 μL of MeCN was added to the mixture, after being incubated under the different conditions described above, for deproteinization. The final nominal concentrations of zonarol were 20, 80, and 320 ng/mL. After deproteinization, the mixtures were centrifuged at 90 rpm for 5 min, and the supernatants were used for HPLC analysis. The accuracy of the assay was determined by comparing the nominal concentrations with the corresponding calculated concentrations via linear regression. Every concentration of zonarol was measured three times.

### 3.7. Plasma Concentration of Zonarol after Oral or Intravenous Administration in Mice

The mice were fasted for 12 h before the oral administration of zonarol. Blood samples were collected from the mouse tails at 15, 30, 45, 60, 90, 120, 240, and 480 min after the oral administration of 100 mg/kg of zonarol (*n* = 5).

The mice were not fasted before the intravenous administration of zonarol but were fasted after the test started. Blood samples were collected from the mouse tails, via the caudal veins, at 5, 15, 30, 45, 60, 90, 120, and 240 min after the intravenous administration of 10 mg/kg of zonarol (*n* = 6).

### 3.8. Tissue Distribution of Zonarol after Intravenous Administration in Mice

Blank tissue homogenates were prepared by collecting the liver, kidney, and brain from normal mice after anesthetization. Blank tissue homogenates were used to determine the specificity of zonarol. In the experimental mice, the livers, kidneys, and brains were collected at 15, 30, 60, 120 min after the intravenous administration, via the caudal veins, of 10 mg/kg of zonarol (*n* = 7). The tissue with added distilled water (mass/volume = 1:4) was homogenized.

Blood samples were immediately centrifuged at 9000 rpm for 5 min. After centrifugation, a 20 μL aliquot of plasma was mixed with 80 μL of MeCN for deproteinization. Then, the mixture was centrifuged at 9000 rpm for 5 min, and the supernatant was used for HPLC analysis.

Next, a 50 μL of tissue homogenate was mixed with 200 μL of MeCN for deproteinization. Then, the mixture was centrifuged at 9000 rpm for 5 min, and the supernatant was used for HPLC analysis.

### 3.9. Data Analysis

Data were expressed as the mean ± standard deviation (SD) for the in vitro and the animal experiments. The number of the data is described for each experiment. The pharmacokinetic parameters were calculated with Phoenix Winnonlin software (version 8.1) while using a non-compartmental model. The abnormal data were eliminated after being analyzed via a Box-type drawing with SPSS software (SPSS Inc., Chicago, IL, USA).

## 4. Conclusions

Zonarol, a marine hydroquinone, is a candidate that has been focused upon due to its various activities, but its properties in vivo have not been sufficiently investigated. To consider its actual applications, it is necessary that its properties are experimentally tested in vivo. In the current report, the sensitive, stable, and simple methods used to detect zonarol in the extracts of biological samples were established via HPLC with a fluorescence detector. The time-course experiments in plasma and tissue were performed in mice after the oral and intravenous administration of zonarol in order to investigate zonarol’s pharmacokinetic properties. A biased distribution of the marine hydroquinone was demonstrated in the brain tissue of zonarol-treated mice. Further research is needed to explain these biased mechanisms and to confirm zonarol’s antibacterial, antioxidative, and anti-inflammatory effects in the target organs for treatment. These current results will be helpful for understanding and estimating the pharmacological effects of zonarol in vivo and its effective dose for any clinical trials that are conducted in the future.

## Figures and Tables

**Table 1:** Repeatability of zonarol detection by HPLC-FI method.

Validation	Zonarol (ng/mL)		RSD (%)	Accuracy (%)
	Prepared	Detected		
intra-day (*n* = 6)	320	309.9 ± 5.06	1.6	96.8
	80	76.7 ± 1.93	2.5	95.9
	20	21.6 ± 0.35	1.6	107.8
inter-day (*n* = 3)	320	306 ± 7.51	2.5	95.6
	80	75.8 ± 0.43	0.6	94.7
	20	21.7 ± 1.14	5.3	108.5

Zonarol concentrations (ng/mL in DMSO) are expressed mean ± SD. Relative standard deviation (RSD) (%) = (SD/mean) × 100. Accuracy (%) = (detected/prepared) × 100.

**Table 2:** Stability of zonarol with plasma during HPLC analysis.

Treatment	Zonarol (ng) vs. 1 mL of Plasma		RSD (%)	Accuracy (%)
	Prepared	Detected		
storage at 4 °C for 12 h	320	334.2 ± 10.70	3.2	104.4
	80	73.8 ± 1.98	2.7	92.3
	20	21.0 ± 1.33	6.4	104.9
storage at RT for 4 h	320	311.1 ± 2.76	0.9	97.2
	80	73.0 ± 1.72	2.4	91.2
	20	22.6 ± 1.24	5.5	112.9
storage at 37 °C for 4 h	320	304.6 ± 6.57	2.0	95.2
	80	72.6 ± 0.53	0.7	90.8
	20	21.2 ± 0.33	1.6	105.8
three cycles of freeze-thaw	320	325.5 ± 4.56	1.4	101.7
	80	75.5 ± 2.38	3.2	94.4
	20	21.6 ± 1.44	6.7	107.9

Zonarol concentrations (ng/mL in plasma) are expressed mean ± SD. Relative standard deviation (RSD) (%) = (SD/mean) × 100. Accuracy (%) = (detected/prepared) × 100.

**Table 3:** The pharmacokinetic parameters of zonarol in mice.

Parameter (unit)	Administration	
	p.o.	i.v.
number of data	*n* = 5	*n* = 6
dose of zonarol (mg/kg)	100	10
C_max_ (ng/mL)	722.51 ± 75.93	—
T_max_ (min)	75 ± 13.41	—
AUC (min × ng/mL)	91,333.84 ± 31,976.29	36,470.64 ± 5734.82
bioavailability (%)	25.0	—

Data are expressed as the mean ± SD. The maximum plasma concentration (Cmax), the time to Cmax (Tmax), and area under curve (AUC) as pharmacokinetics parameters are calculated based on data of time-dependent changing in plasma concentration of zonarol after orally (100 mg/kg, p.o.) and intravenously administration (10 mg/kg, i.v.) as shown in Figure 4A and Figure 4B, respectively. The bioavailability (%) = (p.o. AUC/p.o. dose)/(i.v. AUC/i.v. dose) × 100.

**Figure A1:**
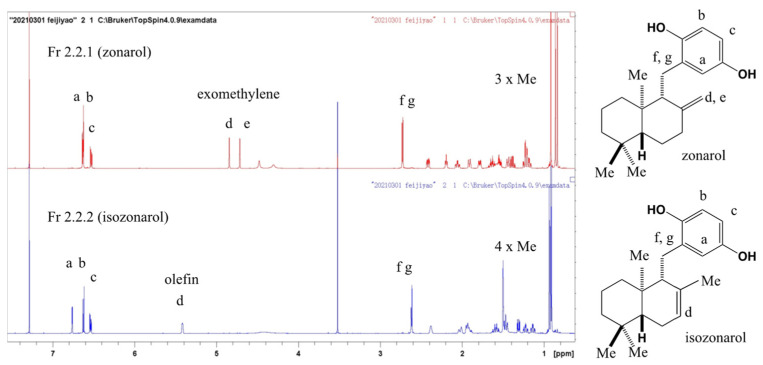
The ^1^H NMR of zonarol and isozonarol (600 MHz, CDCl_3_).

## Data Availability

Not applicable.

## Appendix A. Purification of the Zonarol from the Brown Algae *Dictyopteris undulata*

The frozen algae were freeze-dried (132 g) and powdered with a mill grinder to form an extract with around 20 volumes of methanol (3 L) for 3 days at room temperature. Then, the extract was filtered and evaporated to give a dark brown powder (15.73 g) as the crude extract (100%w). After several partition steps, a 90% MeOH layer (4.38 g) was applied on silica gel in a stepwise elution with Hex/EtOAc. Then, a 1:5 = Hex/EtOAc eluant (1.78 g) was separated on an ODS column with 80% aqueous MeOH to obtain the zonarol-concentrated fraction (1.415 g). Further separation on a Develosil ODS-HG-5 column (i.d. 20 × 250 mm, Nomura Chemical Co., Ltd., Aichi, Japan) was performed and was indicated with a UV at 280 nm with an HPLC system (JASCO Co., Tokyo, Japan). After the final purification step using HPLC with recycle mode on the column, zonarol was obtained as Fr. 2.2.1 (461.8 mg, 2.93%w). And its isomer, isozonarol, as Fr 2.2.2 (371.1 mg, 2.36%w) was also obtained as byproducts. The structure and purity of the purified zonarol were verified via an analysis of the 1D and 2D NMR data, which were measured with an AV-600 (Bruker, Tokyo, Japan). Please see further details in our previous report [16].

## Appendix B. The ^1^H NHR Spectra and Structures of Isolated Zonarol and Isozonarol

The chemical structure and purity of zonarol were confirmed by analyzing the ^1^H-NMR data (600 MHz, CDCl_3_), as is shown in Figure A1. All of the proton signals on the chart corresponded with those of the previous reports [14,32,33]. In addition, the 2D NMR data (COSY, HMQC, and HMBC; however, these data are not shown here) suggested reasonable correlations supporting the structure of zonarol. Although there were no signals derived from impurities on the chart, the purities of the compound were estimated as more than 95%. The purified zonarol was used in all experiments in the current report.

## References

[1] Aneiros A., Garateix A. (2004). Bioactive peptides from marine sources: Pharmacological properties and isolation procedures. J. Chromatogr. B.

[2] Jensen A., Chapman A.R.O., Brown M.T., Lahaye M. (1993). Present and future needs for algae and algal products. Proceedings of the Fourteenth International Seaweed Symposium.

[3] Li Y.-X., Wijesekara I., Li Y., Kim S.-K. (2011). Phlorotannins as bioactive agents from brown algae. Process Biochem..

[4] Kim S.-K., Wijesekara I. (2010). Development and biological activities of marine-derived bioactive peptides: A review. J. Funct. Foods.

[5] Wijesekara I., Kim S.-K. (2010). Angiotensin-I-converting enzyme (ACE) inhibitors from marine resources: Prospects in the pharmaceutical industry. Mar. Drugs.

[6] Wijesekara I., Pangestuti R., Kim S.-K. (2011). Biological activities and potential health benefits of sulfated polysaccharides derived from marine algae. Carbohydr. Polym..

[7] Wijesekara I., Yoon N.Y., Kim S.K. (2010). Phlorotannins from Ecklonia cava (Phaeophyceae): Biological activities and potential health benefits. Biofactors.

[8] Bringloe T.T., Starko S., Wade R.M., Vieira C., Kawai H., De Clerck O., Cock J.M., Coelho S.M., Destombe C., Valero M. (2020). Phylogeny and evolution of the brown algae. Crit. Rev. Plant Sci..

[9] Faulkner D.J. (2001). Marine natural products. Nat. Prod. Rep..

[10] Kang K.A., Kim J.K., Jeong Y.J., Na S.Y., Hyun J.W. (2014). Dictyopteris undulata extract induces apoptosis via induction of endoplasmic reticulum stress in human colon cancer cells. J. Cancer Prev..

[11] Kim A.D., Kang K.A., Piao M.J., Kim K.C., Zheng J., Yao C.W., Cha J.W., Hyun C.L., Boo S.J., Nam Ho Lee N.H. (2014). Dictyopteris undulata extract induces apoptosis in human colon cancer cells. Biotechnol. Bioprocess Eng..

[12] Ishibashi F., Sato S., Sakai K., Hirao S., Kuwano K. (2013). Algicidal sesquiterpene hydroquinones from the brown alga Dictyopteris undulata. Biosci. Biotechnol. Biochem..

[13] Kumagai M., Nishikawa K., Matsuura H., Umezawa T., Matsuda F., Okino T. (2018). Antioxidants from the brown alga *Dictyopteris undulata*. Molecules.

[14] Fenical W., Sims J.J., Squatrito D., Wing R.M., Radlick P. (1973). Zonarol and isozonarol, fungitoxic hydroquinones from the brown seaweed *Dictyopteris zonarioides*. J. Org. Chem..

[15] Mayer A.M.S., Paul V.J., Fenical W., Norris J.N., de Carvalho M.S., Jacobs R.S., Chapman A.R.O., Brown M.T., Lahaye M. (1993). Phospholipase A_2_ inhibitors from marine algae. Proceedings of the Fourteenth International Seaweed Symposium.

[16] Yamada S., Koyama T., Noguchi H., Ueda Y., Kitsuyama R., Shimizu H., Tanimoto A., Wang K.Y., Nawata A., Nakayama T. (2014). Marine hydroquinone zonarol prevents inflammation and apoptosis in dextran sulfate sodium-induced mice ulcerative colitis. PLoS ONE.

[17] Shimizu H., Koyama T., Yamada S., Lipton S.A., Satoh T. (2015). Zonarol, a sesquiterpene from the brown algae *Dictyopteris undulata*, provides neuroprotection by activating the Nrf2/ARE pathway. Biochem. Biophys. Res. Commun..

[18] Han J., Guo X., Koyama T., Kawai D., Zhang J., Yamaguchi R., Zhou X., Motoo Y., Satoh T., Yamada S. (2021). Zonarol protected liver from methionine-and choline-deficient diet-induced nonalcoholic fatty liver disease in a mouse model. Nutrients.

[19] Yankah V.V., Ushio H., Ohshima T., Koizumi C. (1998). Quantitative determination of butylated hydroxyanisole, butylated hydroxytoluene, and tert-butyl hydroquinone in oils, foods, and biological fluids by high-performance liquid chromatography with fluorometric detection. Lipids.

[20] Spencer J.P., Chowrimootoo G., Choudhury R., Debnam E.S., Srai S.K., Rice-Evans C. (1999). The small intestine can both absorb and glucuronidate luminal flavonoids. FEBS letters.

[21] Yan H., Wang L., Li X., Yu C., Zhang K., Jiang Y., Wu L., Lu W., Tu P. (2009). High-performance liquid chromatography method for determination of carnosic acid in rat plasma and its application to pharmacokinetic study. Biomed. Chromatogr..

[22] Satoh T., McKercher S.R., Lipton S.A. (2013). Nrf2/ARE-mediated antioxidant actions of pro-electrophilic drugs. Free Radic. Biol. Med..

[23] Yang L.L., Xiao N., Li X.W., Fan Y., Alolga R.N., Sun X.Y., Wang S.L., Li P., Qi L.W. (2016). Pharmacokinetic comparison between quercetin and quercetin 3-O-β-glucuronide in rats by UHPLC-MS/MS. Sci. Rep..

[24] Kwon S.H., Kang M.J., Huh J.S., Ha K.W., Lee J.R., Lee S.K., Lee B.S., Han I.H., Lee M.S., Lee M.W. (2007). Comparison of oral bioavailability of genistein and genistin in rats. Int. J. Pharm..

[25] Xing J., Chen X., Zhong D. (2005). Absorption and enterohepatic circulation of baicalin in rats. Life Sci..

[26] Lee J., Mitchell A.E. (2012). Pharmacokinetics of quercetin absorption from apples and onions in healthy humans. J. Agric. Food Chem..

[27] Pool H., Mendoza S., Xiao H., McClements D.J. (2013). Encapsulation and release of hydrophobic bioactive components in nanoemulsion-based delivery systems: Impact of physical form on quercetin bioaccessibility. Food Funct..

[28] Zhang Y., Wu H., Wen H., Fang H., Hong Z., Yi R., Liu R. (2015). Simultaneous determination of fucoxanthin and its deacetylated metabolite fucoxanthinol in rat plasma by liquid chromatography-tandem mass spectrometry. Mar. Drugs.

[29] de Boer V.C., Dihal A.A., van der Woude H., Arts I.C., Wolffram S., Alink G.M., Rietjens I.M., Keijer J., Hollman P.C. (2005). Tissue distribution of quercetin in rats and pigs. J. Nutr..

[30] Romo Vaquero M., García Villalba R., Larrosa M., Yáñez-Gascón M.J., Fromentin E., Flanagan J., Roller M., Tomás-Barberán F.A., Espín J.C., García-Conesa M.T. (2013). Bioavailability of the major bioactive diterpenoids in a rosemary extract: Metabolic profile in the intestine, liver, plasma, and brain of Zucker rats. Mol. Nutr. Food Res..

[31] Betsholtz C. (2014). Double function at the blood–brain barrier. Nature.

[32] Cimino G., de Stefano S., Fenical W., Minale L., Sims J.J. (1975). Zonaroic acid from the brown seaweed *Dictyopteris undulate* (which is the same as *zonarioides*). Experientia.

[33] Mori K., Komatsu M. (1986). Synthesis and absolute configuration of zonarol, a fungitoxic hydroquinone from the brown seaweed *Dictyopteris zonarioides*. Bull. Des Soc. Chim. Belg..

